# Supplementary material for: First record of Apanteles hemara (N.) on Leucinodes orbonalis Guenée and biodiversity of Hymenoptera parasitoids on Brinjal
Source: PeerJ. 2024 Mar 29;12:e16870. doi: 10.7717/peerj.16870 (PMC10984170; doi:10.7717/peerj.16870)
Supplement: Supplemental Information 2 [file peerj-12-16870-s002.docx]

**Table S1 Weather parameters during the study period (November 2021 to October 2022) were recorded from an agrometeorological observatory, Division of Agricultural Physics, ICAR-IARI, New Delhi.**

| **Year** | **Month** | **Rainfall** | **Temperature (^°^C)** | | | **Sunshine** | **EP** | **Relative humidity** |
| --- | --- | --- | --- | --- | --- | --- | --- | --- |
|  |  | **(mm)** | **T*_max_*** | **T*_min_*** | **T*_mean_*** | **(hour)** | **(mm)** | **(%)** |
| **2020-21** | **November** | 0.00 | 27.1 | 10.6 | 18.9 | 4.5 | 2.5 | 71.8 |
|  | **December** | 0.31 | 21.7 | 7.2 | 14.4 | 3.8 | 1.7 | 77.9 |
| **2021-22** | **January** | 4.58 | 17.4 | 7.5 | 12.5 | 2.5 | 1.6 | 83.9 |
|  | **February** | 1.07 | 23.1 | 8.7 | 16.0 | 6.5 | 2.8 | 70.6 |
|  | **March** | 0.00 | 32.3 | 15.3 | 23.7 | 8.3 | 4.6 | 59.3 |
|  | **April** | 0.00 | 40.1 | 19.8 | 38.2 | 8.7 | 6.7 | 40.3 |
|  | **May** | 1.94 | 39.8 | 24.8 | 31.8 | 6.9 | 6.9 | 54.9 |
|  | **June** | 2.96 | 40.4 | 26.0 | 33.3 | 6.7 | 6.8 | 50.4 |
|  | **July** | 10.50 | 35.1 | 26.1 | 30.5 | 4.6 | 3.8 | 75.4 |
|  | **August** | 2.56 | 34.1 | 25.6 | 29.4 | 5.5 | 3.8 | 74.2 |
|  | **September** | 6.38 | 34.2 | 24.1 | 29.1 | 5.7 | 4.0 | 76.4 |
|  | **October** | 4.35 | 31.3 | 20.0 | 25.6 | 6.1 | 3.4 | 72.3 |
