# Supplementary figures and images for: First record of Apanteles hemara (N.) on Leucinodes orbonalis Guenée and biodiversity of Hymenoptera parasitoids on Brinjal
Source: PeerJ. 2024 Mar 29;12:e16870. doi: 10.7717/peerj.16870 (PMC10984170; doi:10.7717/peerj.16870)

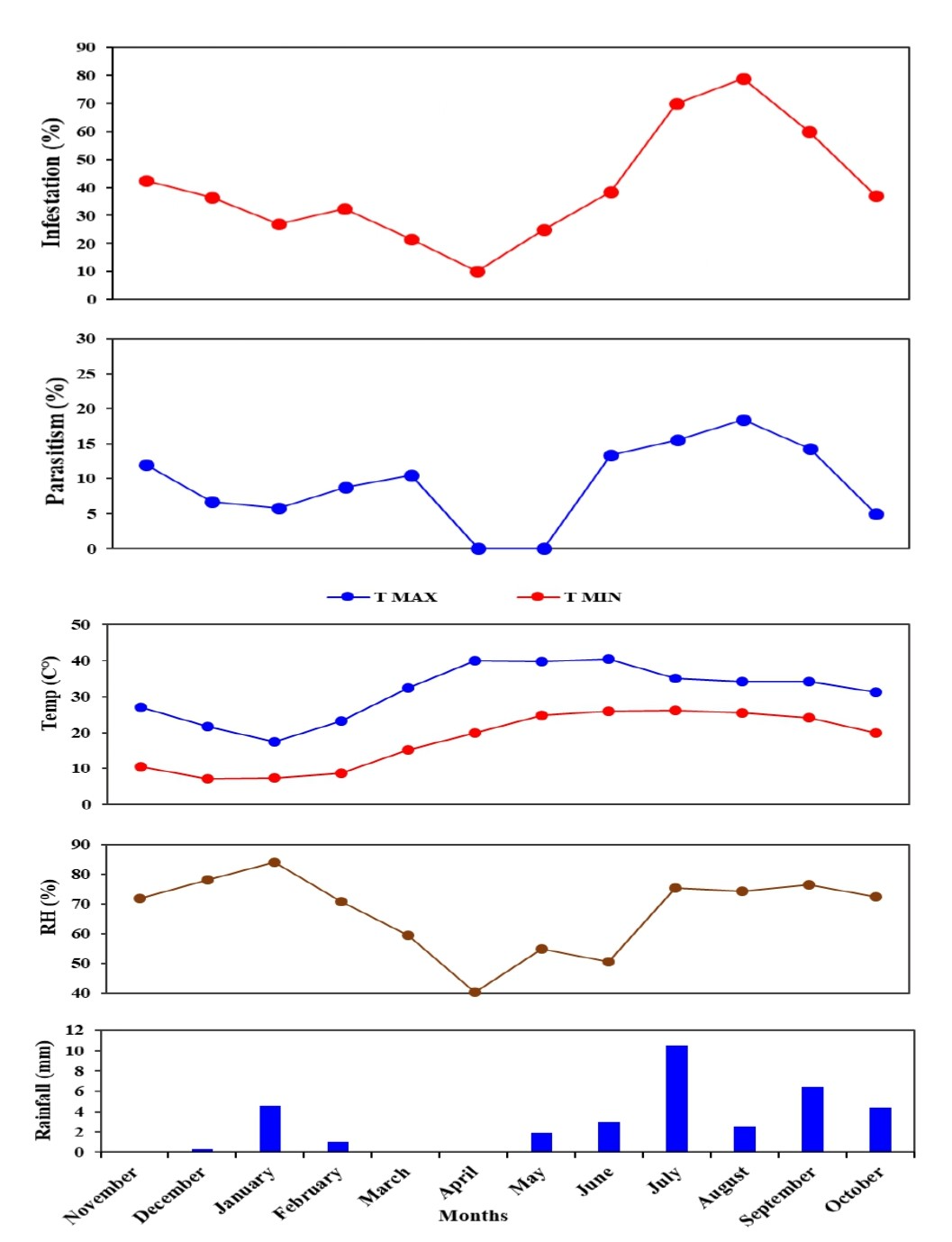

Supplement: Supplemental Information 3 [file peerj-12-16870-s003.png]

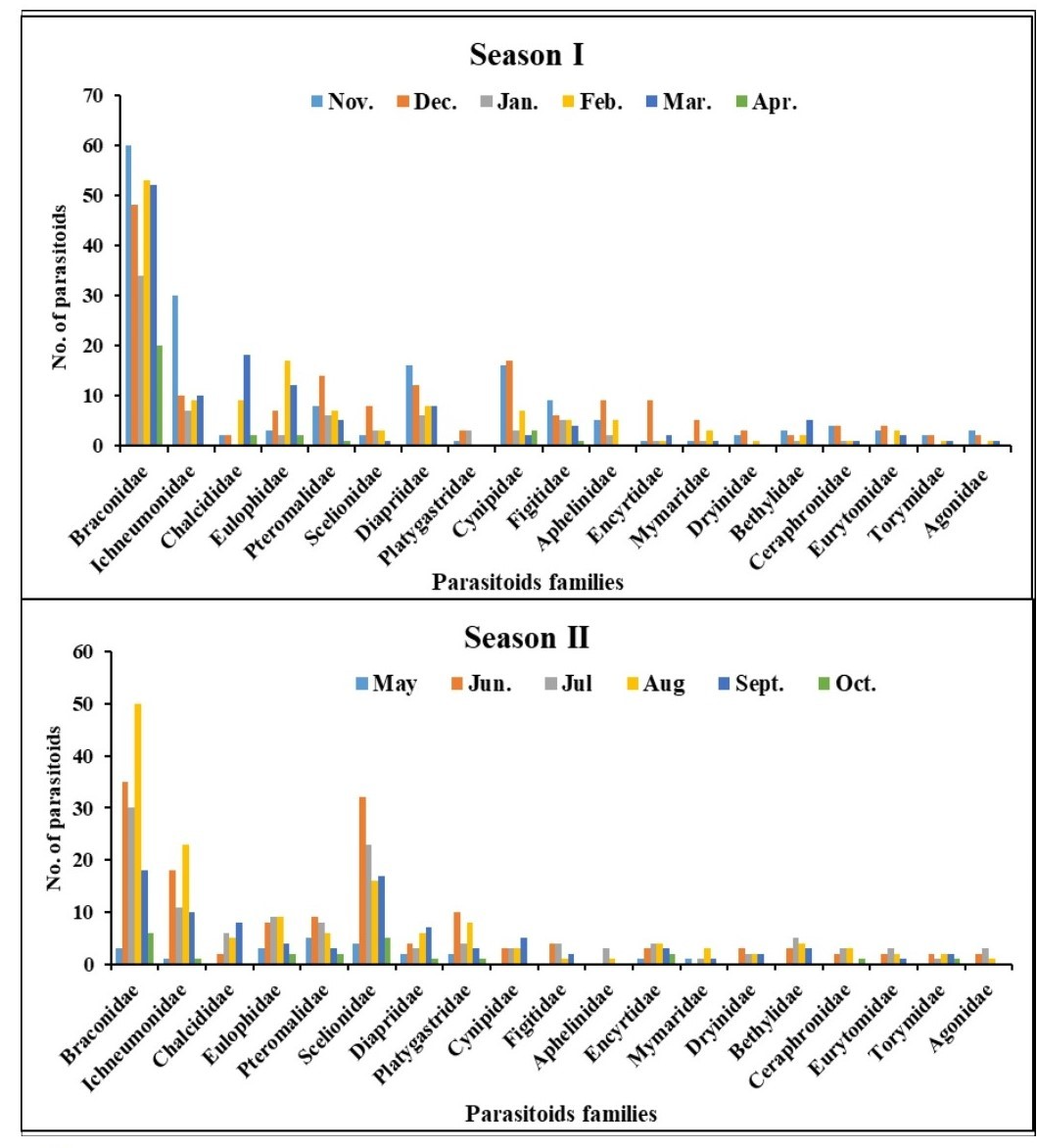

Supplement: Supplemental Information 4 [file peerj-12-16870-s004.png]
